# The impact of circulating protein levels identified by affinity proteomics on short-term, overall breast cancer risk

**DOI:** 10.1038/s41416-023-02541-2

**Published:** 2023-12-22

**Authors:** Felix Grassmann, Anders Mälarstig, Leo Dahl, Annika Bendes, Matilda Dale, Cecilia Engel Thomas, Marike Gabrielsson, Åsa K. Hedman, Mikael Eriksson, Sara Margolin, Tzu-Hsuan Huang, Mikael Ulfstedt, Simon Forsberg, Per Eriksson, Mattias Johansson, Per Hall, Jochen M. Schwenk, Kamila Czene

**Affiliations:** 1 Department of Medical Epidemiology and Biostatistics, Karolinska Institutet, Stockholm, Sweden; 2 Institute for Clinical Research and Systems Medicine, Health and Medical University, Potsdam, Germany; 3 Pfizer Worldwide Research, Development and Medical, Stockholm, Sweden; 4 Science for Life Laboratory, Department of Protein Science, KTH Royal Institute of Technology, Solna, Sweden; 5 Department of Oncology, Södersjukhuset, Stockholm, Sweden; 6 Department of Clinical Science and Education Södersjukhuset, Karolinska Institutet, Stockholm, Sweden; 7 Cancer Immunology Discovery, Pfizer Inc., San Diego, CA USA; 8 Olink Proteomics, Uppsala Science Park, Uppsala, Sweden; 9 Genomic Epidemiology Branch, International Agency for Research on Cancer (IARC/WHO), Lyon, France

**Keywords:** Predictive markers, Risk factors, Epidemiology

## Abstract

**Objective:**

Current breast cancer risk prediction scores and algorithms can potentially be further improved by including molecular markers. To this end, we studied the association of circulating plasma proteins using Proximity Extension Assay (PEA) with incident breast cancer risk.

**Subjects:**

In this study, we included 1577 women participating in the prospective KARMA mammographic screening cohort.

**Results:**

In a targeted panel of 164 proteins, we found 8 candidates nominally significantly associated with short-term breast cancer risk (*P* < 0.05). Similarly, in an exploratory panel consisting of 2204 proteins, 115 were found nominally significantly associated (*P* < 0.05). However, none of the identified protein levels remained significant after adjustment for multiple testing. This lack of statistically significant findings was not due to limited power, but attributable to the small effect sizes observed even for nominally significant proteins. Similarly, adding plasma protein levels to established risk factors did not improve breast cancer risk prediction accuracy.

**Conclusions:**

Our results indicate that the levels of the studied plasma proteins captured by the PEA method are unlikely to offer additional benefits for risk prediction of short-term overall breast cancer risk but could provide interesting insights into the biological basis of breast cancer in the future.

## Introduction

Breast cancer is the most common cancer in women worldwide, with incidence rates still increasing in Western countries. While recent advances in therapy have increased the odds of survival after a breast cancer diagnosis, early detection of aggressive breast cancer is paramount to further improve health in our aging population. Current mammographic screening programmes have a number needed to screen around 1000–2000 [1, 2], indicating many women have to be screened every 2 years for 10 years to save a single life. Thus, our current screening programmes need to be improved by better detection of women at risk of developing invasive breast cancer, particularly within the next screening interval.

Traditional risk prediction algorithms are mainly based on reproductive risk factors, genetic risk factors such as aggregate genetic risk scores, family history and lifestyle factors. More recent efforts to identify women with a high short-term or long-term risk for breast cancers used clinical models that additionally included features from mammographic images such as breast density or the presence of microcalcifications [3, 4]. Those models have shown high discriminatory performance compared to traditional risk models and are now suitable for identifying individuals at high risk for breast cancer. Nevertheless, the sensitivity and specificity of the models can potentially be further improved by identifying additional (independent) risk factors.

To this end, there are several approaches to identifying novel (molecular) markers for breast cancer risk. Current large-scale efforts focus on genome-wide scans to identify genetic factors that influence the overall and/or subtype-specific breast cancer risk [5–8]. In addition, other molecular markers such as DNA modifications [9], circulating metabolites [10], and cell-free DNA/RNA [11], as well as proteins [12, 13] are being studied. Apart from inherited genetic markers, only a few other biomarkers have been successfully validated in independent studies [14]. In addition, many past and currently underway studies suffer from several limitations, such as small sample size and lack of available incident cases not confounded by treatment [15].

In this study, we present the results from the KARMA cohort, the largest prospective breast cancer screening cohort in Sweden. We measured plasma protein levels in an exploratory and a targeted panel and analysed their association with incident breast cancer to identify novel markers for breast cancer risk.

## Methods

### Study population

Women aged 40–74 years are invited every 18–24 months to the national screening programme in Sweden. Women attending the mammographic screening in two regions (Stockholm and Skåne) in Sweden were invited to participate in the KARMA study between 2011 and 2013. A total of 70,877 women gave informed consent to participate in the KARMA study [16]. Participants answered a comprehensive web-based questionnaire, donated blood, and accepted linkage to national registers. From linkage to the cancer registry, we identified 826 women diagnosed with breast cancer which occurred within 3 years of blood draw between 2012 and 2015. From those, only 804 had plasma specimens and thus were used in our study. We used the *matchit* function from the *MatchIt* library implemented in R to match 804 controls from KARMA study to the incident cases by randomly drawing women without incident breast cancer so that the median age at blood draw in cases and controls was similar (median matching).

We used self-reported questionnaire data to create dichotomous variables for menopausal and smoking status. Family history of first-degree relatives was assessed from the multi-generation registry, as previously described [17]. BMI and age were assessed at the time of study entry and thus at the time of qualifying blood draw. Linkage to the prescription registry was used to determine whether women had taken a lipid medication (ATC code C10) between 2005 and blood draw.

Tumour characteristics such as oestrogen receptor (ER) status, human epidermal growth factor receptor 2 (HER2) status, grade and lymph node involvement were retrieved from medical records or from the Swedish National Cancer Registry. Mode of detection was defined by the timing between the last mammographic screening and time of diagnosis [18, 19]. Briefly, women diagnosed between two scheduled screening intervals without a detectable tumour in the previous screening were deemed to have interval breast cancer (IC). Conversely, women diagnosed with breast cancer at a regularly scheduled mammogram are considered screen-detected (SDC). Patients who did not attend screening or missed their scheduled screening prior to diagnosis were not considered in the analysis of IC vs. SDC (Supplementary Table 1).

### Protein measurements—targeted panel

The samples from the Karma cohort were distributed across 96-well plates with samples from the same individual placed on the same plate and the remaining samples randomly distributed. Samples from Skåne and Stockholm were placed on separate plates. Proximity Extension Assay was performed at SciLifeLab’s Affinity Proteomics Unit in Stockholm according to instructions from Olink Proteomics AB (Uppsala, Sweden) [20] to measure proteins in EDTA plasma using the Cardiometabolic (v.3603, Lot No A94923) and Immuno-Oncology (v.3111, Lot No B01401) panels. For the cardiometabolic panel, plasma samples were diluted 1:2025, and for the immune-oncology panel 1:1 (undiluted). Normalised protein expression (NPX) values were obtained from the Olink NPX Manager software (version 2.2.1.311) after normalisation using the “Intensity normalisation v2” method to account for the various measurement batches [21]. In addition to standard quality control measures, we removed proteins that had missing values in more than 10% of the samples, either in the Skåne or the Stockholm recruitment centre. After quality control, 163 high-quality protein measurements from the Cardiometabolic and Immuno-Oncology panel were available for 796 incident cases and 781 controls (Table 1) from both cohorts. As an additional quality control maker, we also computed the percentage of proteins that were below the level of detection (LOD) in each participant and recorded the duration the plasma was stored at −80 °C (age of plasma). To account for differences between the protein levels by recruitment centre, we used a rank-based inverse normal transformation on each protein in both cohorts separately with the *qnorm* function in R. From this normalised data, we used the *prcomp* function in R to compute the first ten principal components (PCs) to capture additional underlying data structures represented by those PCs.

**Table 1:** Summary statistics of included KARMA participants at baseline exam.

	Stockholm		Skåne	
Variable	Controls	BC cases	Controls	BC cases
Number of individuals	410	405	371	391
Mean age (SD) [years], matched*	58.48 (9.56)	58.52 (9.60)	58.66 (9.82)	59.05 (9.66)
Mean body mass index (SD) [kg/m^2^]	25.16 (4.19)	25.61 (4.20)	25.33 (4.25)	25.76 (4.13)
Postmenopausal [%]	70.49	69.38	70.35	72.38
Ever smoked [%]	58.00	59.17	50.70	55.59
Lipid medication taken [%]	11.46	11.85	12.40	15.60
Hypertensive medication taken [%]	27.07	22.72	28.30	26.09
Heart medication taken [%]	11.71	10.86	11.86	9.46
Renal failure (prevalent) [%]	0.73	0.25	0.54	0.00
Mean age of plasma [years]	7.92 (0.65)	7.53 (0.71)	8.02 (0.70)	7.61 (0.76)
Average frequency of proteins below LOD (SD)	0.12 (0.02)	0.12 (0.02)	0.14 (0.02)	0.14 (0.02)
Mean 313 SNP Genetic Risk Score (SD)	−0.08 (0.29)	−0.02 (0.30)	−0.07 (0.32)	−0.05 (0.31)

*LOD* level of detection.

^*^Variable used for median matching cases and controls.

### Protein measurements—exploratory panel

Similar to the approach for the targeted panel of proteins, we also measured over 3000 proteins from eight different panels with a Proximity Extension Assay (Olink Proteomics AB, Uppsala, Sweden) in a subset of individuals from the Skåne cohort. The raw protein measurements were analysed with the Olink NPX Manager software as described above to yield normalised protein expression values. Proteins with more than 50% missing values were removed from analyses as were those flagged with a warning or error from the NPX Manager software. The less stringent cut-off for the exclusion of proteins was chosen since the exploratory panel contains many proteins only present in minute concentrations and thus can often be below level of detection across the cohort. Furthermore, individuals that were flagged as outliers by principal component analyses were also excluded, yielding a final analytical dataset consisting of 2204 proteins in 303 BC cases and 294 controls (for more details, see ref. [22]). Similar to the small panel, we also computed the first principal components from the protein data and used those as additional exposures in our association analyses.

### Breast cancer genetic risk score

All cases and controls were genotyped on the OncoArray genotyping platform and passed standard quality control, as previously described [18]. Briefly, we excluded related individuals, individuals with excessive missingness (> 3% missing sites before variant QC) or heterozygosity as well as individuals not of European descent. In addition, variants with a strong deviation from Hardy–Weinberg equilibrium (*P* < 0.00001) or with a high degree of missingness (missing in more than 10% of individuals) were also excluded. For more details, please see refs. [18, 22].

Breast cancer risk was quantified by a genetic (polygenic) risk score from 313 variants associated with breast cancer risk as previously described with *plink2* [7]. Briefly, the genotype at each variant (coded as the number of risk-increasing alleles 0, 1 or 2) was multiplied with the respective log odds ratio indicating the strength of association with breast cancer risk. Then, for each individual, the weighted alleles were summed up over all variants, yielding a single score for each individual. Higher genetic risk scores indicate a higher genetic risk for breast cancer from common variants, while lower scores indicate lower risk.

### Statistical analysis and presentation

Unsupervised clustering of the raw NPX values by their Euclidean distance was performed with the *heatmap.2* function from the *gplot* package [23]. All association analyses were carried out with Cox proportional hazard models, as implemented in the *survival* package in R [24]. We adjusted the models for known confounders of protein levels or breast cancer risk. In particular, the models were adjusted for age at blood draw, BMI, lipid and heart medication, renal failure, smoking status, menopause status, family history of breast cancer, breast density, age of plasma (i.e., duration of storage), number of proteins below LOD, the 313 variant genetic risk score and, where appropriate, recruitment centre. The results of the association analyses were plotted as Manhattan plots with the *ggplot* function implemented in the *ggplot2* library [25] or as correlation plots with the *corrplot* function implemented in the *corrplot* library [26].

## Results

### Quality control and unsupervised clustering

**Fig. 1:**
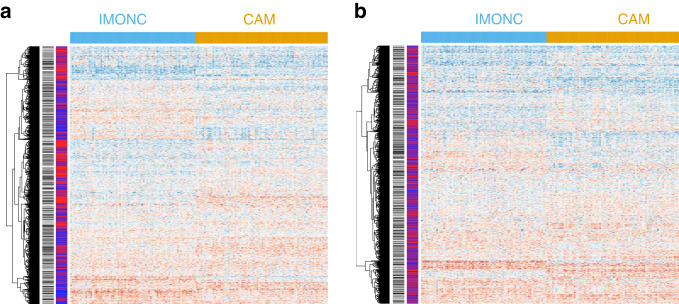
Unsupervised clustering of patients and controls according to protein levels. **a** Cases and controls are clustered according to recruitment centre (Skåne in blue and Stockholm in red). **b** After quantile normalisation of protein levels of participants from each centre, no striking differences between centres were obvious. Future disease status is indicated by black (BC) and white (control) bars. IMONC Immuno-Oncology (v.3111) panel, CAM Cardiometabolic (v.3603) panel.

After quality control, a total number of 163 proteins from the Olink Proximity Extension Assay (PEA) Cardiometabolic and Immuno-Oncology panels (targeted panel) were available for analysis in 796 incident cases and 781 controls, which were selected from two cohorts recruited in Stockholm and in the Skåne region in Sweden, respectively (Table 1 and Supplementary Table 1). Baseline characteristics ascertained at blood draw (Table 1) as well as tumour characteristics (Supplementary Table 1) were similar among both cohorts. First, we performed an unsupervised clustering approach and observed that individuals were broadly grouped according to the recruitment centre in which they were recruited (Fig. 1a). To account for this, we normalised protein levels within each region by computing a rank-based inverse normal transformation. This effectively removed the systematic difference in protein levels observed by the recruitment centre (Fig. 1b). The remaining clusters were not representative of other covariates nor of disease status (Fig. 1b and Table 1).

### Association of proteins with breast cancer risk in the targeted and exploratory panel

**Fig. 2:**
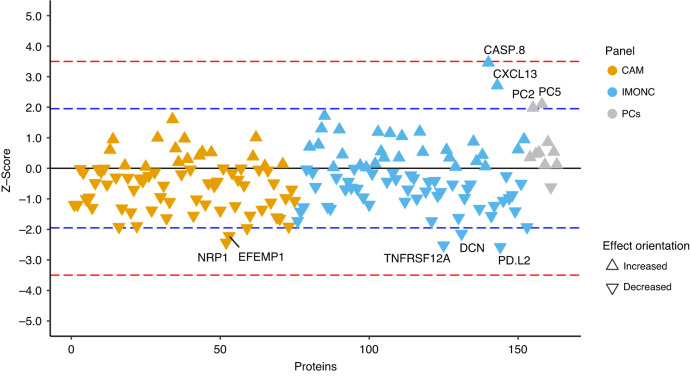
Association of 163 proteins in the targeted panel with incident breast cancer. We used Cox proportional hazard models to investigate the impact of cardiometabolic (CAM) and immune-oncology (IMONC)-related proteins on the risk to develop breast cancer within 3 years. Triangles pointing up indicate that increased protein levels result in increased risk for breast cancer and vice versa. Significantly associated proteins (*P* < 0.05) are shown above or below the blue dotted line and are labelled. No proteins were found to be significantly associated with incident breast cancer risk after accounting for multiple testing (i.e., *P* < 0.05/163), as indicated by the red dotted line.

Next, we computed the association of normalised plasma protein levels in the targeted panel with breast cancer risk using Cox regression while adjusting for potential confounders such as age at blood draw, BMI, smoking status, renal failure, family history of breast cancer, the 313 simple nucleotide polymorphism (SNP) genetic risk score for breast cancer and medication. In the targeted panel, we found 8 proteins nominally associated with breast cancer risk (*P* < 0.05), of which two proteins showed a positive and six a negative effect size (Fig. 2). Notably, among the significantly associated proteins was Caspase 8 (CASP8), which has previously been implicated in breast cancer genetic risk as well as other cancers. However, after adjustment for multiple testing (either by controlling the false discovery rate, FDR, at FDR < 0.05 or Bonferroni correction), none of the proteins remained statistically significant (Fig. 2). We did not find a significant association of the principal components computed from the protein data with breast cancer after adjustment for multiple testing (Fig. 2).

**Fig. 3:**
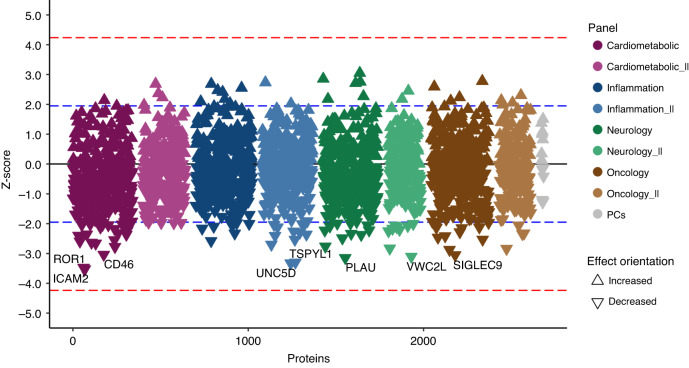
Association of 2,204 proteins in the exploratory panel with incident breast cancer. We used Cox proportional hazard models to investigate the impact of over 2204 proteins on the risk to develop breast cancer. Triangles pointing up indicate that increased protein levels result in increased risk for breast cancer, while triangles pointing down signify lower risk. Significantly associated proteins (*P* < 0.05) are shown above or below the blue dotted line. No proteins were found to be significantly associated with incident breast cancer risk after accounting for multiple testing (i.e., *P* < 0.05/2204), as indicated by the red dotted line.

Considering the complexity and scale of the circulating proteome, it remained a possibility that none of the 163 proteins analysed on the targeted Olink Cardiometabolic and Immune-oncology panels represented proteins of relevance for breast cancer risk. Therefore, we measured a total of 2950 plasma proteins using the Olink Explore I and II panels in a subset of the Skåne study only to avoid confounding the analysis by the recruitment centre (Supplementary Table 2). After quality control, 2204 proteins remained for statistical analysis in 303 incident breast cancer cases and 294 controls. We performed the association analyses in those sample with the same adjustments as before. Here, 115 proteins were nominally associated with breast cancer risk (Fig. 3). However, none of the 115 proteins nor the first ten principal components survived Bonferroni multiple testing correction (*P* < 0.05/2,204, Fig. 3) nor were they significant at FDR < 0.05.

To gain further insights into the nominally significantly associated proteins (*P* < 0.05) from the targeted panel, we investigated the association of those proteins with breast cancer risk after stratifying the patients according to their tumour characteristics (Supplementary Table 1). In general, the proteins are associated similarly in women with different prognostic markers (Supplementary Fig. 1). For particular markers, we see that it is associated more pronounced with more aggressive prognostic markers. Indeed, CXCL13 is statistically significantly more strongly associated with interval compared to screen-detected cancer in a case-only analysis (*P* = 0.005). In addition, CASP8 seems to be more strongly associated with less favourable markers, although it is not significantly different in a case-only design comparing unfavourable against favourable markers (i.e., BC cases with ER-negative, lymph node-positive or high-grade tumours compared to women with ER-positive, lymph node-negative and low-grade tumours).

### Lasso regression

**Fig. 4:**
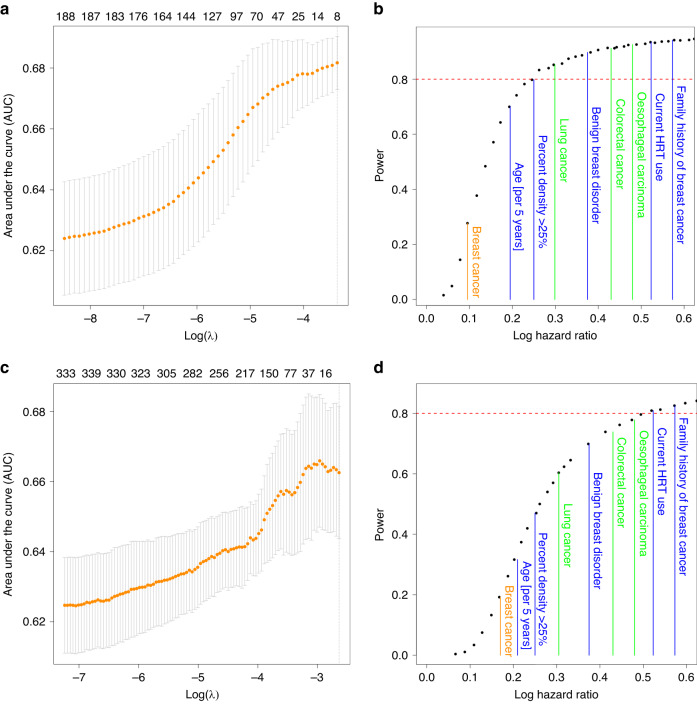
Lasso regression and power analysis of the targeted and exploratory panel. **a** (targeted), **c** (exploratory) We used lasso regression to quantify the potential impact of proteins on the accuracy to predict incident breast cancer. While accounting for (and thus not regularising) known breast cancer risk factors, we computed the area under the receiver operating curve (AUC) for each model using a fivefold cross-validation. The mean AUC as well as the standard error of the AUC estimate from the five cross validations is plotted against the penalty parameter λ and the number of proteins/parameters in the model (numbers at the top). Generally, adding proteins to the model did not improve prediction accuracy. **b** (targeted), **d** (exploratory) The power to detect proteins significantly associated with BC was estimated from generating random data with distributions similar to the observed data. By artificially increasing the effect size, we estimated at which effect size we would have had a power of 80% (red line) to detect significant effects (i.e., observed a Bonferroni corrected *P* value below 0.05). The effect sizes of known risk factors for breast cancer are indicated in blue. In green, we highlighted the average absolute effect sizes observed for nominally significant proteins (*P* < 0.05) for other cancers such as Oesophageal Squamous Cell Carcinoma [36], Colorectal Cancer [36] and Lung Cancer [37] from previous publications. The average absolute effect size observed for proteins with *P* < 0.05 in our dataset for Breast Cancer are shown in orange.

While we did not find that proteins were significantly associated with breast cancer risk individually, they could still exhibit combinatorial effects that would help to predict a future breast cancer diagnosis. To this end, we used Lasso regression to detect whether a combination of proteins in addition to established risk factors would improve risk prediction accuracy, as assessed by the area under the receiver operating curve (AUC, Fig. 4a, c). In this analysis, we found that the baseline model containing age at blood draw, BMI, percent mammographic density, menopause status, the 313 BC genetic risk score and family history of breast cancer outperformed models with additional protein level measurements in the targeted (Fig. 4a) and in the exploratory panel (Fig. 4c). This finding strongly indicates that the studied proteins are not useful for risk prediction of overall, short-term breast cancer risk.

### Power considerations

Next, we studied whether the lack of association with breast cancer risk could potentially be attributed to a lack of power. Although this study is the largest prospective study investigating the association of proteins with incident breast cancer by a large margin, we could still be underpowered to detect such associations. Therefore, we generated random subsets of our data and artificially inflated the effect sizes of the association of each protein with breast cancer. This allowed us to compute the post hoc power of our study to identify specific proteins with a significant *P* value after accounting for multiple testing (i.e., Bonferroni corrected *P* value < 0.05). We found that we had more than adequate power (> 80%) to detect proteins that were associated with breast cancer risk with a hazard ratio greater than 1.28 per standard deviation (SD, log hazard ratio per SD >0.25, Fig. 4b) in the targeted panel, which is more than adequate to identify risk factors with effect sizes observed for benign breast disease or elevated breast density. Similarly, even though we only measured the exploratory panel in less than half of our cohort, we still had sufficient power to detect 80% of all associations with a hazard ratio above 1.74 (per SD, Fig. 4d), which corresponds to effect sizes observed for proteins associated with oesophageal carcinoma, or established risk factors such as hormone replacement therapy and family history. These results mean that we would have ample power to detect associations previously reported for other cancers. However, the effect sizes we observed for nominally significantly associated proteins (*P* < 0.05) was 0.10 and 0.18 on average for the targeted and the exploratory panel, respectively. Those findings show that none of the investigated proteins are likely associated with overall breast cancer risk with large and thus relevant effect sizes as observed in previous studies for other cancers.

## Discussion

In this study, we present results from the largest study to date about circulating proteins involved in breast cancer risk. We found that neither the pre-selected proteins in the targeted panel nor proteins from the extended Olink panel were significantly associated with short-term general breast cancer risk. The lack of associations was not due to low power but attributable to the small effect sizes observed for even nominally significant proteins. Based on our results, we cannot exclude a role of plasma proteins in breast cancer risk but their impact on risk is likely to be low and thus they are of little value to improve risk prediction efforts.

Interestingly, we found that CASP8 was nominally associated with a short-term risk for breast cancer providing insights into a potential role of plasma CASP8 levels in breast cancer risk. Several in vitro studies have shown that CASP8 is involved in apoptosis and necroptosis [27] in different cell types. Inherited genetic variations in the *CASP8* gene have also previously been found to be associated with breast cancer [28] as well as other types of cancers [29, 30]. Importantly, circulating plasma levels of this protein seem to influence the risk for prostate [31] and oesophageal squamous cell carcinoma (ESCC) [32] as well as type 2 diabetes [33] and coronary events [34]. Given its role in diverse phenotypes and involvement in multiple pathways leading to apoptosis, the observed association with breast cancer is still not easily understood. Thus, additional studies designed to provide mechanistic insights into the precise role of CASP8 or related genes and downstream targets in breast cancer risk are needed. Similar to genome-wide association studies, which generally identify variants weakly associated with disease risk, our results are encouraging that high-throughput protein panels can potentially identify important proteins and thus pathways in breast cancer even if individual proteins only show weak associations.

Previous studies using Olink protein panels have often found stronger effect sizes than those we observed in our cohort for nominally significant proteins [31, 32, 35–37]. This could be attributed to multiple factors, such as smaller sample size in preceding studies or the winner’s curse effect, both of which would strongly bias and inflate the estimates, as much as twice their true value [38]. However, even though those previous studies reported exaggerated effect sizes, we would still have enough power to pick up even signals with smaller impact. Conversely, the effect sizes we observed in our cohort are much smaller than most established risk factors, indicating that protein levels are not strongly associated with general breast cancer risk. This is in agreement with the results from the lasso regression, which showed that the addition of proteins does not improve risk prediction accuracy beyond a baseline model containing established hormonal, reproductive, lifestyle, and family history/genetic risk markers. Thus, the proteins we studied are not only weakly associated with breast cancer risk, but they appear unlikely to be informative for future efforts to predict overall breast cancer risk.

Our results do not preclude that proteins might be useful in predicting certain types of breast cancer, either defined by its aggressiveness (i.e., grade and lymph node involvement) or by cell surface markers (such as oestrogen receptor or human epidermal growth factor receptor 2 (HER2) status) [39]. Indeed, we found that the association strength can differ by breast cancer subtype (see Supplementary Fig. 1), although we only observed a statistically significant difference in the association signals for CXCL13 with interval cancer vs. screen-detected cancer. Therefore, larger studies of incident breast cancer cases would be necessary to include a sufficient number of cases with rarer but highly relevant characteristics, such as triple-negative tumours and those that spread beyond the breast. In addition, a combination of proteins and established or yet unknown risk factors could help to predict breast cancer risk in an individualised fashion for certain subtypes of breast cancer. Such efforts, however, require even more extensive studies and, importantly, independent replication in incident breast cancer cases. Such efforts could be possible by including data from individuals recruited in the UK Biobank, which is currently measuring the 3000 proteins in >50,000 randomly selected individuals [40]. Given the incidence of breast cancer in the UK Biobank of around 700 women per year since recruitment, this would translate to around 140 incident breast cancer patients with such protein measurements within 2 years since recruitment. Hence, our study is still at least twice as large and therefore more powered to detect associations with short-term risk, which would be most useful for risk prediction in current screening programmes.

Many of the circulating proteins targeted by the PEA approach originate from organs involved in metabolic or inflammatory processes predominantly due to active secretion [41]. In addition, the proteins can also be potentially contained in diverse extracellular vesicles (EVs) to facilitate intercellular communication, immune response, blood coagulation, and tissue repair [42]. Alternatively, the proteins can originate from an (undetected) tumour which either actively or passively secretes those proteins. Our approach, however, is not suited to easily distinguish between those processes. When we stratified our breast cancer patients by time between blood draw and diagnosis according to the median (in this case, 19 months), we found similar associations in both groups, indicating that leakage of proteins from the tumour is unlikely to be the main driver of the blood proteome in breast cancer captured by our PEA approach. Thus, detecting cancer risk-related proteins originating from the tumour, remains a known challenge if these are not enhanced by systemic involvement of metabolic or inflammatory processes. Compared to classical proteomics platforms using mass spectrometry, the chosen affinity-based PEA assay enables highly sensitive analysis of low-abundant blood proteins [43]. However, there remain relevant functional protein characteristics involved in governing human health [44] that were not resolved with this approach such as the presence of different proteoforms [45] and interacting proteins [46]. These protein traits may still harbour information relevant to breast cancer risk but remain out of reach when analysing systemic blood fluid.

In conclusion, our results indicate that the levels of the investigated proteins captured by a Proximity Extension Assay are unlikely to be informative to improve risk prediction of short-term breast cancer risk. Still, our study was designed to study overall breast cancer risk. Thus, a study focused on breast cancer subtypes (i.e., tumour characteristics or survival) could identify more specific associations which could prove useful for predicting certain types of breast cancer. Finally, despite low effect sizes observed in our dataset, we note that protein data can potentially be leveraged in future studies to gain important insights the biology underlying breast cancer, thus enabling the identification of novel preventive targets.

### Supplementary information

Supplementary Material

## Data Availability

Access to phenotypes, biospecimens and genotypes from the KARMA study can be requested from https://karmastudy.org/contact/data-access/.
