# Supplementary material for: The impact of circulating protein levels identified by affinity proteomics on short-term, overall breast cancer risk
Source: Br J Cancer. 2023 Dec 22;130(4):620–7. doi: 10.1038/s41416-023-02541-2 (PMC10876928; doi:10.1038/s41416-023-02541-2)

**Supplementary Table 1. Mode of detection and tumor characteristics for KARMA breast cancer (BC) cases**

|  | **KARMA BC Cases** | |
| --- | --- | --- |
| **Variable** | **Skåne** | **Stockholm** |
| Number of individuals | 391 | 405 |
| Screen-detected BC [%] | 65.47 | 70.37 |
| Interval BC [%] | 31.46 | 24.44 |
| Missing mode of detection [%] | 3.07 | 5.18 |
| ER positive [%] | 85.42 | 88.64 |
| Missing ER status [%] | 1.28 | 2.22 |
| HER2 positive [%] | 11.25 | 10.62 |
| Missing HER2 status [%] | 2.30 | 4.20 |
| Grade I or II [%] | 62.92 | 67.90 |
| Grade III [%] | 28.90 | 27.65 |
| Missing Grade [%] | 8.18 | 4.44 |

|  | **Skane** | |
| --- | --- | --- |
| **Variable** | **BC Cases** | **Controls** |
| Number of individuals | 303 | 294 |
| Mean age (S.D) [years], matched* | 58.76 (9.50) | 59.26 (9.25) |
| Mean body mass index (S.D) [kg/m2] | 25.73 (4.16) | 25.23 (4.18) |
| Postmenopausal [%] | 71.95 | 74.15 |
| Ever smoked [%] | 55.94 | 52.33 |
| Lipid medication taken [%] | 15.84 | 10.88 |
| Hypertensive medication taken [%] | 24.42 | 26.53 |
| Heart medication taken [%] | 8.91 | 9.86 |
| Renal failure [%] | 0.00 | 2.04 |
| Mean age of plasma [years] | 7.65 (0.76) | 8.06 (0.75) |
| Average frequency of proteins below LOD (S.D) | 0.04 (0.02) | 0.04 (0.02) |

**Supplementary Table 2. Summary statistics of KARMA participants at baseline exam used in the exploratory protein panel**

* Variable used for median matching cases and controls

LOD = Level of detection

**Supplementary Figure 1. Association of plasma proteins with breast cancer risk stratified by tumor characteristics.**We used Cox regression to estimate the association between levels of nominally significantly associated proteins (P<0.05, see **Figure 2**) and risk for breast cancer. Cases were stratified by the tumor characteristics (**Supplementary Table 1**) of BC women and compared to controls. The strength of the association (log hazard ratio from the Cox regression models) is shown in colour and by the size of the circles. Statistically significant associations (P<0.05) are indicated with an asterisk. Low grade = Grade I or II; High Grade = Grade III. ER = Estrogen receptor status


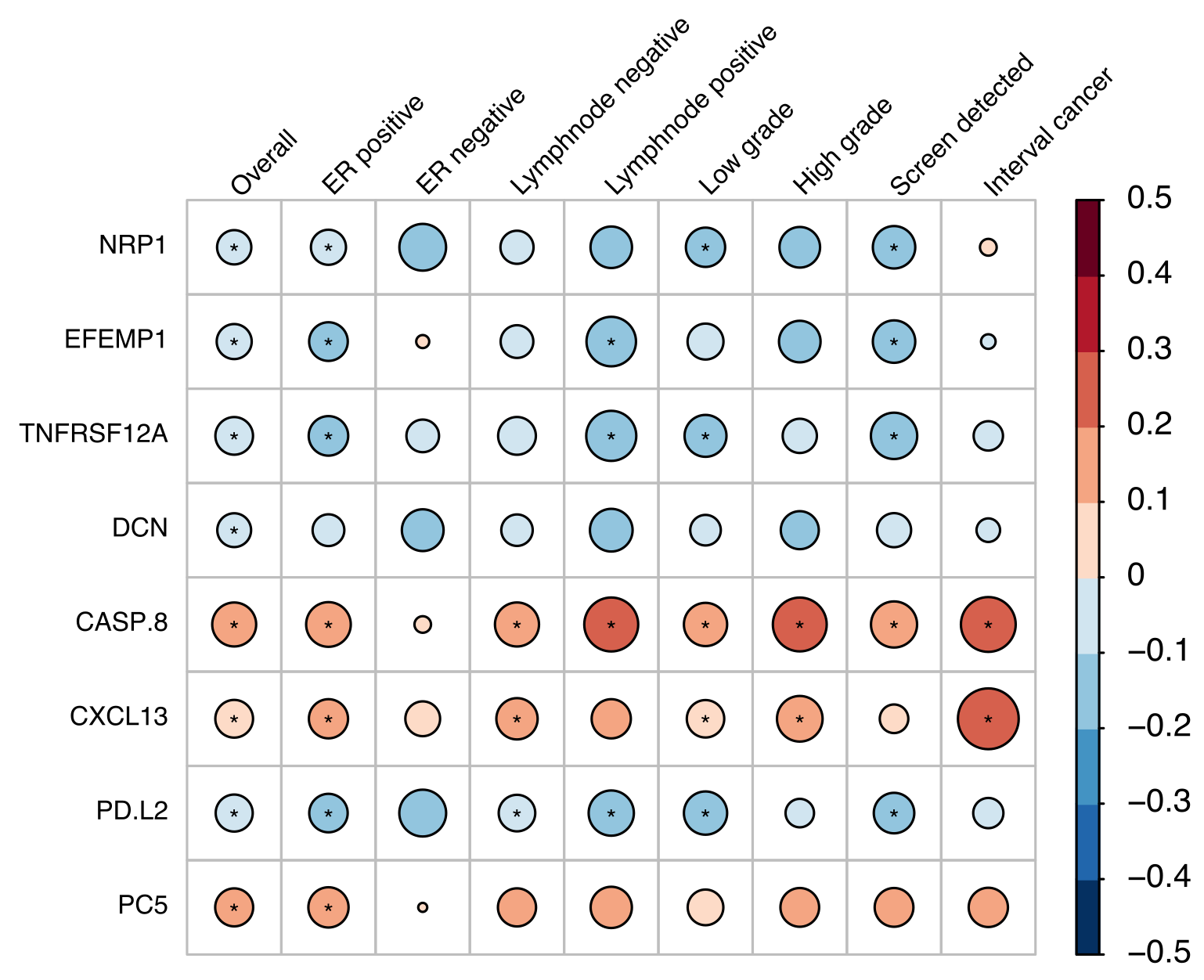

Supplement: Supplementary file 1 — Supplementary Material [file 41416_2023_2541_MOESM1_ESM.docx]
